# Anomalous Right Superior Vena Cava in an Asymptomatic Patient

**DOI:** 10.7759/cureus.8265

**Published:** 2020-05-24

**Authors:** Hardik Bhatt, Megan McGreevy, Charles J Chung, Sharma Kattel

**Affiliations:** 1 Internal Medicine, University at Buffalo, Buffalo, USA; 2 Pediatric Cardiology, Oishei Children's Hospital, Buffalo, USA; 3 Radiology, State University of New York, Buffalo, USA; 4 Cardiology, University at Buffalo, Buffalo, USA

**Keywords:** anomalous svc, adult congenital heart disease, absent right superior vena cava, azygos vein, congenital heart disease

## Abstract

Congenital superior vena cava (SVC) anomalies are not uncommon. However, an absence of a left SVC and an anomalous right SVC without additional congenital heart defects is very rare. We present a 38-year-old male with an 'anomalous SVC' that was found to be descending anterior to the pleural space and draining into the inferior vena cava (IVC) at the level of the right atrium. This was associated with an anomalous right upper and lower pulmonary vein draining into this anomalous SVC. To our knowledge, this combination of congenital anomalies has not been previously described in the medical literature.

## Introduction

Congenital systemic venous anomalies have been well documented in the medical literature. Specifically, anomalies of the superior vena cava (SVC) have been commonly found in asymptomatic patients. The most common anomaly of the SVC is the persistence of a left-sided SVC, seen in about 10% of patients with congenital heart disease and 0.5%-2% of the general population [1]. Additionally, the presence of a left-sided SVC in the absence of a right-sided SVC has been found to be in 0.09%-0.13% of the population [2]. Bilaterally absent SVCs is a much rarer anomaly. The first reported case was in 1981, discovered during a transvenous pacemaker placement for complete atrioventricular block and diagnosed via venography [3]. In this initial case, along with subsequent cases of bilaterally absent SVCs, the superior venous drainage was generally split amongst azygos veins and collaterals traveling along the midline [3-5].

Here, we present an individual who was found to have bilaterally absent SVCs with an ‘anomalous SVC’ descending anterior to the pleural cavity along the midclavicular line and draining at the inferior vena cava (IVC) at the level of its insertion with the right atrium. To our knowledge, this combination of congenital anomalies has not been previously documented.

## Case presentation

**Figure 1:**
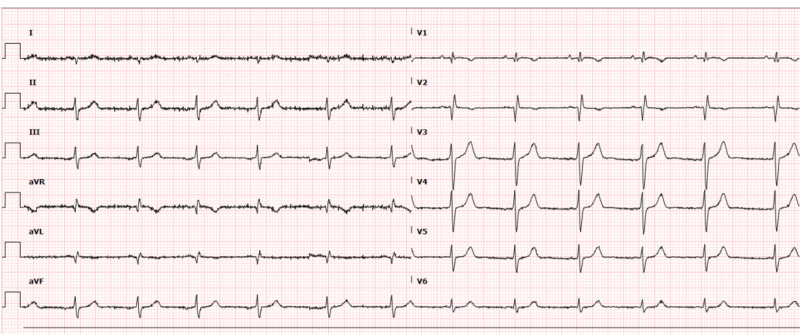
Initial electrocardiogram revealing an ectopic atrial rhythm, right axis deviation, right ventricular hypertrophy and an incomplete right bundle branch block pattern.

A 38-year-old male with no significant past medical history presented to an urgent care with complaints of cough, fatigue and decreased appetite for the past five days. Initial evaluation with a chest X-ray (CXR) was concerning for a right-sided lung mass, and he was subsequently referred to the emergency department for further evaluation. Although his symptoms of fatigue and decreased appetite were already resolving, he decided to seek medical attention for a continued non-productive cough. Initial physical exam was unremarkable, and his basic laboratory work was within normal limits with the exception of a positive influenza A on a rapid enzyme-linked immunosorbent assay (ELISA) test. An electrocardiogram (ECG) done on admission revealed an ectopic atrial rhythm, right axis deviation, right ventricular hypertrophy and an incomplete right bundle branch block pattern (Figure 1). 

**Figure 2:**
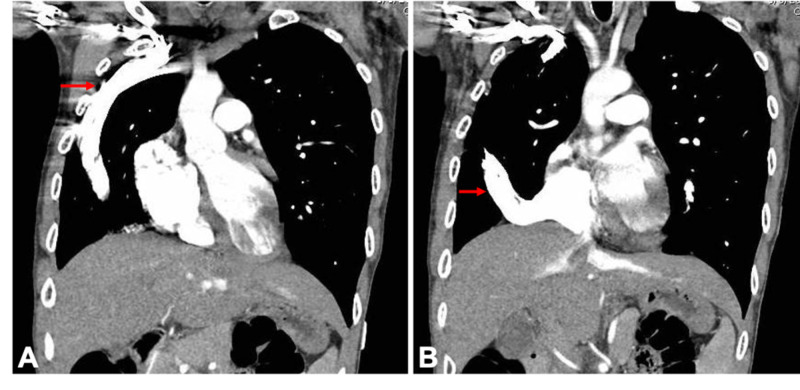
CT angiogram of the chest (coronal sections). (A) Superior portion of the large anomalous superior vena cava draining the right jugular, subclavian and innominate vein that extends caudally in the anterior pleural space. (B) Inferior portion of the same anomalous superior vena cava communicating with the inferior vena cava at the junction of the right atrium.

**Figure 3:**
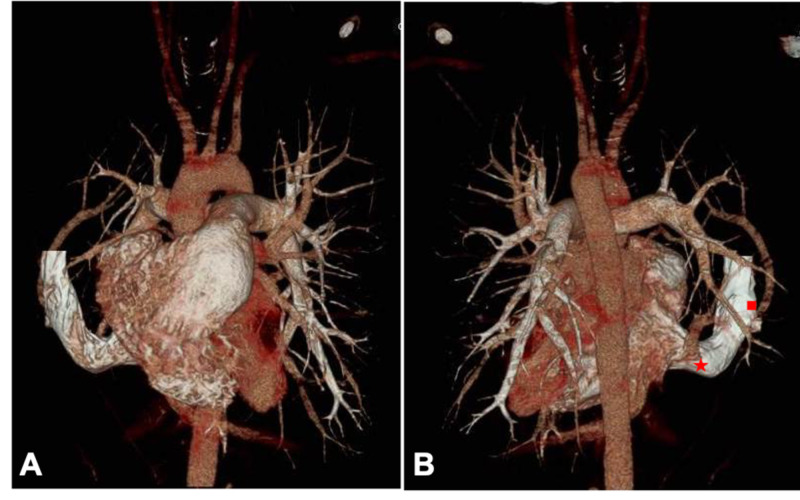
A three-dimensional reconstructed CT scan image of the heart. (A) The anterior view of the anomalous superior vena cava inserting into the right atrium with the inferior vena cava. (B) The posterior view of the anomalous superior vena cava. The large right lower lobe anomalous pulmonary vein (star) and right upper lobe pulmonary vein (square) can be seen inserting into the anomalous superior vena cava.

Subsequent evaluation of this possible right lung mass with a contrast-enhanced computed tomography (CT) angiogram of the chest showed no evidence of a lung mass or a pulmonary embolism but rather anomalies of the venous vasculature. There was a large anomalous SVC receiving drainage from the right jugular, subclavian vein and possibly the innominate vein, extending caudally in the anterior pleural space (Figure 2A), subsequently descending medially and communicating with the intrathoracic IVC at the junction of the IVC and right atrium (Figures 2B, 3A). A prominent left intercostal vein was visible, but a large left-sided SVC was not appreciated. Additionally, a large right lower lobe anomalous pulmonary vein was draining into the anomalous SVC at the junction with the IVC and a right upper lobe pulmonary vein was also found to be draining into the anomalous SVC proximal to the IVC/right atrial junction (Figure 3B). The right atrium and right ventricle were enlarged, and the pulmonary artery appeared to be dilated. Additionally, mild left ventricular hypertrophy was present, but no large atrial or ventricular septal defects were appreciated.

## Discussion

The development of the primordial venous system in the embryo occurs around the sixth week of gestation. Initially, the cardinal veins form the main venous drainage system of the embryo. The bilateral anterior cardinal veins drain the cranial portions of the embryo, whereas the bilateral posterior cardinal veins drain the caudal portions of the embryo. Around the eighth week, the anterior cardinal veins can be found splitting to form the subclavian and jugular veins, bilaterally. Additionally, during this time an oblique anastomosis forms between the left and the right anterior cardinal veins. This shunt ultimately becomes the left brachiocephalic vein and the subsequent caudal part of the left anterior cardinal vein obliterates and becomes ‘the ligament of Marshall’. The right anterior cardinal vein and common cardinal vein then form the SVC.

Venous vascular malformations are relatively not that uncommon. The most common is the persistence of a left-sided SVC or bilateral SVCs, which form when the caudal portion of the left anterior cardinal vein does not obliterate. In patients with a left-sided SVC, venous drainage into the right atrium is via the coronary sinus. The venous return in patients found to have bilaterally absent SVC is much more variable. In prior case reports, patients with bilaterally absent SVCs were found to have superior venous drainage through the azygos veins traveling along the midline and inserting into the IVC below the level of the diaphragm [3-5]. This can be explained by the obliteration of bilateral common cardinal veins and subsequent venous flow into the supracardinal veins which eventually form the azygos and hemiazygos veins. Both veins were then found to have communications with the IVC further downstream, within the abdominal cavity.

**Figure 4:**
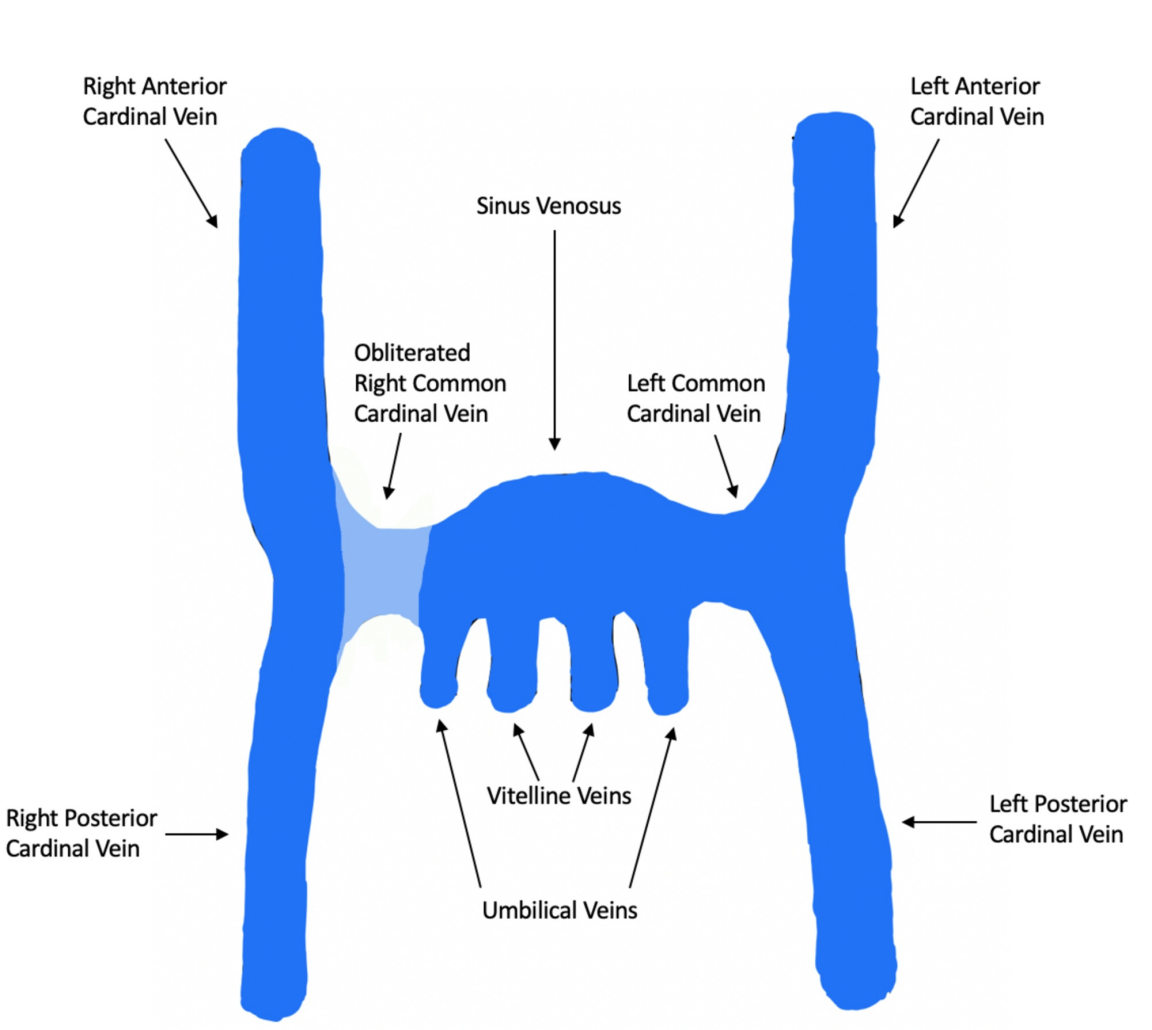
Illustration of the primordial veins of an embryo at six weeks of gestation. The right common cardinal vein is shown to be obliterated preventing the normal communication between the right anterior cardinal vein and the sinus venosus.

In our patient, an azygos vein was not present and the anomalous vein tracked caudally, anterior to the pleural space and then medially back toward the right atrium. The embryologic basis for this malformation is not quite clear. But based on the origins of the primordial venous vessels, we postulate that the right-sided common cardinal vein likely obliterated which prevented the communication between the right anterior cardinal vein and the sinus venosus, thereby preventing the formation of a right-sided SVC (Figure 4). Instead, the continuous high pressure led to the persistence of the right posterior cardinal vein which then canalized along the midline eventually coursing medially and inserting into the thoracic IVC. The persistence of the right posterior cardinal vein likely inhibited the formation of the right-sided supracardinal vein, which normally becomes the azygos vein.

The embryologic development of the pulmonary veins begins as solitary vessels within the mediastinal tissues, which are distinct from the embryonic systemic venous sinus [6]. These vessels then canalize blood from the pulmonary vein plexus and use the regressing dorsal mesocardium as a portal of cardiac entry [6]. In this patient, the right-sided pulmonary veins did not extend into the left atrium but instead formed an anastomosis with the anomalous SVC. This is likely due to the close proximity to the anomalous vessel within the right mediastinum.

A similar condition called Scimitar syndrome has been well documented in the literature. In patients with this syndrome, an anomalous right pulmonary vein or the ‘Scimitar vein’ drains most, if not all, of the right lung into the IVC. Imaging results in these patients reveal a large anomalous vein traveling along the midline of the right lung field along with dextroposition of the heart, hypoplasia of the right lung field and hypoplasia of the right pulmonary artery [7]. However, in our patient, there was no significant hypoplasia of the right lung, the heart was relatively normal in position and the anomalous vein communicated superiorly with the right jugular, subclavian and innominate vein not consistent with features of Scimitar syndrome.

Fortunately, our patient did not have sequela of congenital heart disease as a child and has remained fairly asymptomatic so far, but he has started to show some signs of right ventricular dilation. This warrants further quantification of his left-to-right shunt and potential surgical correction to avoid the dreaded complication of right heart failure and subsequent Eisenmenger syndrome. These individuals with large venous anomalies pose significant challenges when there is a need for right ventricular pacemaker lead insertions, electrophysiology procedures, systemic venous cannulation for cardiopulmonary bypass, endomyocardial biopsies and partial or total cavopulmonary anastomoses [8]. Clear understanding of the patient’s vasculature is essential prior to any of these procedures. Furthermore, these individuals also pose a significant risk for the development of sinus node dysfunction and/or heart block [9]. Therefore, it is imperative to follow these patients regularly.

## Conclusions

Cases of congenital anomalies of the SVC are not uncommon in the medical literature. But the presence of an anomalous right SVC that descends anterior to the pleural cavity and drains into the right atrium at the level of the IVC has not been previously found. Additionally, this patient was found to have anomalous pulmonary veins that inserted into the anomalous SVC, thereby creating a left-to-right shunt. Our patient remained asymptomatic when this anomaly was found, but he continues to be at significant risk for the development of right heart failure and Eisenmenger syndrome. These vascular abnormalities also complicate any future cardiothoracic procedures or surgeries. These patients should have prompt evaluation of their vasculature with an echocardiogram and cardiac catherization to determine the need for early intervention. 
